# COVID-19 early detection for imbalanced or low number of data using a regularized cost-sensitive CapsNet

**DOI:** 10.1038/s41598-021-97901-4

**Published:** 2021-09-16

**Authors:** Malihe Javidi, Saeid Abbaasi, Sara Naybandi Atashi, Mahdi Jampour

**Affiliations:** 1 Quchan University of Technology, Quchan, Iran; 2 Ferdowsi University of Mashhad, Mashhad, Iran; 3 Department of Radiology, Shariati Hospital, Tehran University of Medical Sciences, Tehran, Iran

**Keywords:** Computed tomography, Computer science

## Abstract

With the presence of novel coronavirus disease at the end of 2019, several approaches were proposed to help physicians detect the disease, such as using deep learning to recognize lung involvement based on the pattern of pneumonia. These approaches rely on analyzing the CT images and exploring the COVID-19 pathologies in the lung. Most of the successful methods are based on the deep learning technique, which is state-of-the-art. Nevertheless, the big drawback of the deep approaches is their need for many samples, which is not always possible. This work proposes a combined deep architecture that benefits both employed architectures of DenseNet and CapsNet. To more generalize the deep model, we propose a regularization term with much fewer parameters. The network convergence significantly improved, especially when the number of training data is small. We also propose a novel Cost-sensitive loss function for imbalanced data that makes our model feasible for the condition with a limited number of positive data. Our novelties make our approach more intelligent and potent in real-world situations with imbalanced data, popular in hospitals. We analyzed our approach on two publicly available datasets, HUST and COVID-CT, with different protocols. In the first protocol of HUST, we followed the original paper setup and outperformed it. With the second protocol of HUST, we show our approach superiority concerning imbalanced data. Finally, with three different validations of the COVID-CT, we provide evaluations in the presence of a low number of data along with a comparison with state-of-the-art.

## Introduction

Without any doubt, the pandemic COVID-19 is the most crucial event in 2020, which started at the end of 2019 and it continues. When writing this study, some companies claimed to provide its vaccine, but new variants of the virus have emerged that the effect of the vaccine on these species is unknown and needs further study; however, extensive efforts in this area are promising. Therefore, traditional awareness and early detection are essential that are offered to affect its treatment dramatically. However, its early detection is challenging due to its pandemic and the massive number of affected people. Nevertheless, the good news is that the very recent deep learning (DL) technology can significantly help physicians recognize if a person is affected with coronavirus^1,2^. Deep learning is a branch of machine learning and aims to learn like humans by implementing brain neural networks’ straightforward structure. Such technology relies on the taken chest CT scan as a choice modality for evaluating lung involvement and analysis for detection of COVID-19 pneumonia^3,4^.

In recent years several artificial deep neural networks (DNNs) were introduced as a powerful tool for solving image-related problems^5^, including medical image analysis and COVID-19 pneumonia classification^6,7^. For instance, ResNet^8^, DenseNet^9^, CapsNet^10^, SENet^11^ are some of the very successful DNNs that provide significant learning and analyze the problems similar to humans. Nevertheless, a significant drawback of these artificial DNNs is the need for much training data. Unlike humans who can intelligently understand and inference the facts, these artificial DNNs need to analyze data to understand. Therefore, the lack of appropriate data may occur overfitting when the model learns just a few specific system behaviors. Consequently, it is not general enough. To this end, several approaches used popular data augmentation or proposed to employed Generative Adversarial Networks (GAN)^12^, or its family such as Cycle-GAN (CGAN)^13^, etc. to generate similar data artificially.

The challenge of data is essential for real-world problems due to the unavailability or difficulty of preparing relevant data. For instance, in COVID-19 pneumonia, early detection of the disease and correct diagnosis in the thousands of chest CT images need adequate expert physicians for decisions in an ideal condition that is not simply feasible. On the other hand, imbalanced training data, which is very common in real-world problems, is another gap for such artificial DNNs. For instance, again, in COVID-19 pneumonia, there are tens of thousands of chest CT images before the presence of Coronavirus in 2019 that could be useful for negative training samples. Still, in contrast, we currently have a very limited number of positive samples of CT images. This imbalanced data can negatively affect the current artificial deep neural networks.

### Novelty and contributions of the study

**Figure 1:**
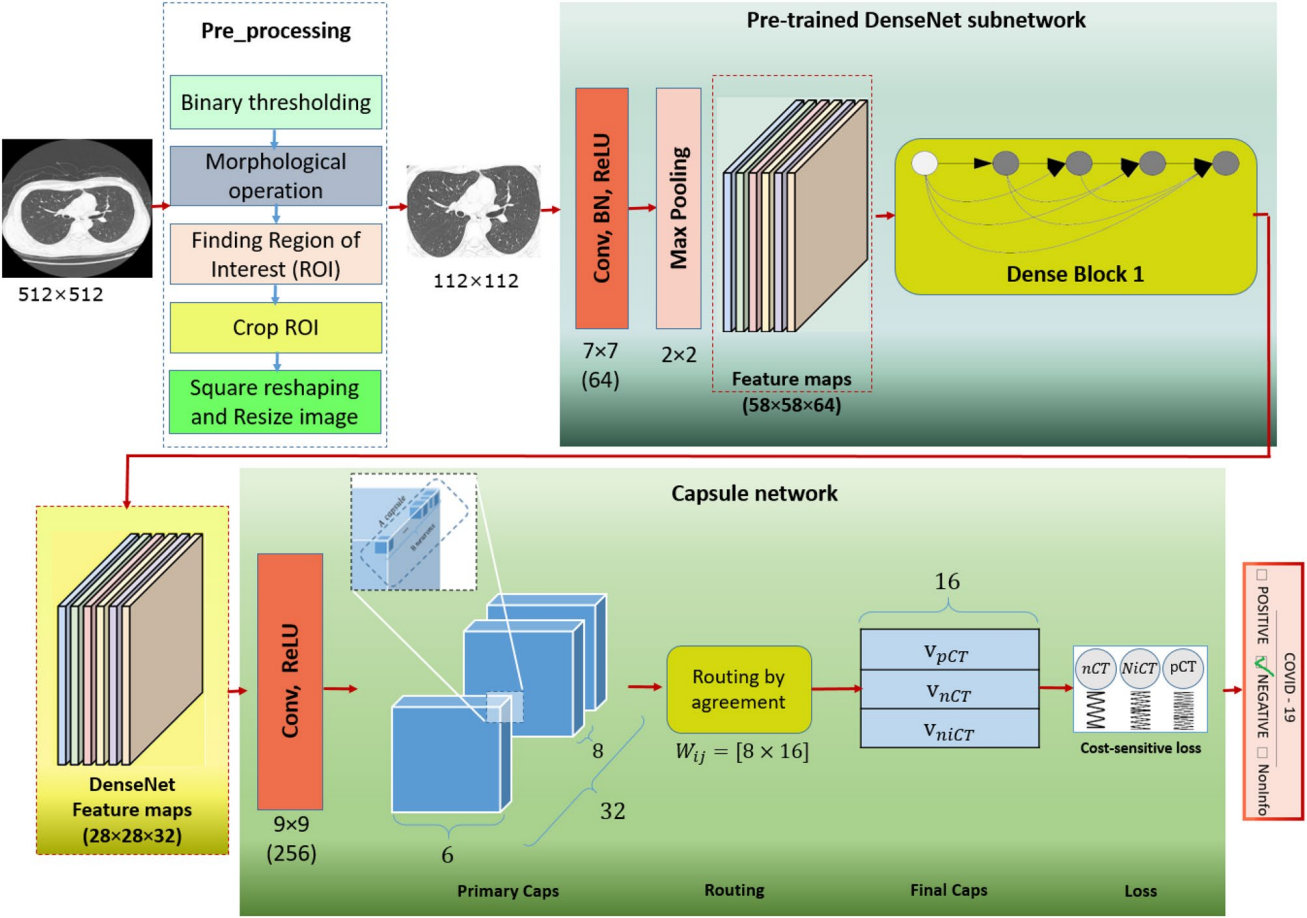
Our regularized cost-sensitive CapsNet combined with DenseNet (RegCS.CapsDensNet) overall architecture. CT image is first preprocessed and then feed into our deep network, where its crucial features are extracted by DenseNet conjugating with CapsNet. The cost-sensitive regularized loss function is considered in the capsule network to overcome imbalanced data.

In this study, we propose a hybrid deep neural network for the COVID-19 pneumonia early detection, which is shown in Fig. 1. As a contribution, our model is an efficient hybrid architecture of CapsNet and DenseNet that benefits both deep networks advantages. In addition, our first novelty is proposing a Regularized Cost-sensitive loss of a hybrid network that leads to handling imbalanced data. The second novelty relies on proposing an enhanced regularization function for CapsNet that makes our approach capable of recognizing the COVID-19 in the presence of a low number of training data. To show our approach superiority, we evaluate it on two well-known and publicly available datasets with different protocols.

### Related work

Very recent and successful approaches reported by^14,15^ reveal that DL can provide COVID-19 pneumonia early recognition using CT images. For instance, Di et al.^14^ extract regional and radiometric features from CT image then formulate the relationship among the known COVID-19 cases via a hypergraph structure. Finally, a vertex-weighted hypergraph learning mechanism is used to predict a new case is COVID-19 or not. In this direction, the RT-PCR is an advised diagnostic method for COVID-19 pneumonia. Nevertheless, chest CT scan is a relevant, rapid, accessible, and helpful modality as an alternative screening method^16^. In another work, Matteo et al.^17^ proposed a light CNN which benefits from a low prediction time for COVID-19 detection from CT images. COVID-19 pneumonia shows typical and atypical chest CT scan manifestations. The most common presentations are multilobar patchy peripherally located airspace consolidations and ground-glass opacities. As we know, recognition of chest CT scan involvement patterns is helpful in rapid diagnosis and treatment^18^. In some high suspicion patients for COVID-19 pneumonia, the initial RT-PCR test may be negative despite positive chest CT scan findings. Therefore the role of imaging modalities and the need for rapid diagnostic access is essential to isolate patients^19^. DL techniques are diagnostic methods with high sensitivity and specificity to identify COVID-19 patients as soon as possible^20^. For instance, an explainable (clearly understandable to human users) deep neural network (xDNN) is proposed in^21^. Unlike the general DNNs, xDNN explains its efficiency in terms of time and computational resources. Similar manifestations between COVID-19 pneumonia and other types of pneumonia are challenging, and deep learning is an accurate diagnostic method for differentiating such cases^22^. Due to the busy work of physicians and the high load of involvement with patients, the role of DL as an adjunct method in diagnosis is valuable, and deep learning methods, based on the single chest CT image, demonstrate high diagnostic accuracy for COVID-19 pneumonia^23,24^.

The rest of the paper is organized as follows. Section “Method” introduces the foundation architecture and our motivation to employ DenseNet and CapsNet. The Proposed Framework, DenseNet Combination with regularized Cost-sensitive CapsNet, is presented in the following. In “Results”, we provide extensive evaluations on two datasets with various protocols. Our discussions, including the limitations and upcoming works, are presented in “Discussion and future works”. In the end, we conclude our study.

## Method

### The foundation architectures

For our hybrid architecture, we employed two well-known and successful deep architectures of DenseNet and CapsNet. It has been previously shown that the DenseNet is an efficient architecture in comparison with lots of other deep networks such as VGGNet, ResNet, Inception, SqueezeNet, etc., on the same problem of COVID-19 detection^7,25,26^. In the following, we describe our employed networks and propose then our hybrid architecture.

#### The DenseNet

The DenseNet^9^ is a very successful Deep Neural Network (DNN) in recent years that achieved appropriate results in various tasks. It can be considered as an extended architecture of ResNet, which is known as a prosperous DNNs. Unlike the ResNet, which uses shortcut connections to carry the input data into the next blocks, the DenseNet exploits the impact of shortcut connections for further blocks. This exploitation is such a way that the input image caring to the next blocks and the outcome of the previous blocks are similarly passed to the following blocks. This architecture provides some valuable benefits, such as alleviating vanishing-gradient, which is similar to the ResNet, encouraging the reuse of features, and strengthening feature propagation. At the same time, its number of parameters is efficiently reduced.

#### The CapsNet

Capsule network (CapsNet) is another well-known deep network that proposes to model hierarchical relationships. Using the idea of preserving hierarchical pose relationships, the CapsNet attempts to more mimic biological neural organization. Such a high-level understanding is not straightforward because this built-in perception of an object is not designed inside the other networks. However, the hazardous point in the CapsNet is that the capsule consists of a collection of neurons with instantiation parameters denoting expressed by activity vectors. These components encapsulate all essential information about the state of the feature they are detecting in vector form. The length of an output capsule represents the probability that the feature is present in the current input. Also, the vector’s directions encode some internal state of the detected object^10^.

In the traditional CNNs, the scalar-output features are learned during backpropagation. However, these features are replaced with vector form in the capsule and multiplied by weight matrices to produce prediction vectors. More precisely, suppose each capsule *i* (where $1 \le i \le n$) in layer *l* has an activity vector $u_i \in R^d$ where *d* is the dimension of the primary capsules which encodes the hierarchical relationships. This vector is fed into all capsules *j* in the next layer $l + 1$ and multiplies by a corresponding weight matrix $W_{ij}$ as Eq. (1), to obtain a prediction vector ${\hat{u}}_{j|i}$.

1 $$\begin{aligned} {\hat{u}}_{j|i} = W_{ij} u_i \end{aligned}$$

A weighted sum of all these prediction vectors is calculated to obtain $s_j$, shown by Eq. (2). The iterative dynamic routing algorithm determines these weights called routing coupling coefficients:

2 $$\begin{aligned} s_j = \sum _i c_{ij} {\hat{u}}_{j|i} \end{aligned}$$

To make sure that the capsule’s length is enforced to be no more than 1, the nonlinear squashing function takes the vector $s_j$ and produces the output $v_j$ for all higher-level capsules as Eq. (3).

3 $$\begin{aligned} v_j = \frac{\left\| s_j \right\| ^2}{1+\left\| s_j \right\| ^2} \frac{s_j}{\left\| s_j \right\| } \end{aligned}$$

### Our proposed framework

While it has been shown that a mixture of deep architectures can perform improvement on COVID-19 detection^27,28^, this section proposes an end-to-end deep architecture model by an efficient combination of CapsNet and DenseNet. Also, we propose to enhance our architecture using a Cost-sensitive loss function and an efficient Regularization. The detail is presented in the following.

#### DenseNet combination with CapsNet (DCC)

As explained in the previous section, the CapsNet is a successful shallow network that can effectively extract features with their position relationship. It can provide a high-level understanding of part-whole hierarchy in an object useful for detecting COVID-19 pathology, which usually starts around the lungs. In addition, it has already been shown that it is an efficient architecture with limited training data^29^. Besides, as explained before, the DensNet is a very efficient deep structure with excellent feature extraction and representation. Since the feature extraction of CapsNet is not rich enough, we propose to utilize DenseNet due to its efficient feature extraction and fed them to the capsule network. On the other hand, it has been previously shown that the combination of two deep networks can achieve the advantages of both networks^30^. Inspired by this idea, we propose a new architecture from CapsNet and DenseNet to accomplish the position relationship understanding of CapsNet and feature representation of DenseNet. To this end, as shown in Fig. 1, we propose to train DenseNet and extract features of the second block that are neither considered low-level nor high-level features but including desirable information. Therefore, in our architecture, we obtain the feature maps with $28 \times 28$ and 32 channels and feed them as input data to the capsule network, which applies 256 convolution kernels. In the following, we propose enhancing our combined architecture with efficient regularization and cost-sensitive loss function.

#### Regularized cost-sensitive CapsNet with DenseNet (RCCD)

This section proposes to create a novel and very efficient regularized Cost-sensitive deep neural network (DNN) architecture. Our cost-sensitive loss makes our model capable of handling imbalanced data. It is an essential need in real-world problems such as our application on COVID-19 early detection due to positive data limitation. Therefore, with our model, we can analyze similar problems that have imbalanced data. Moreover, we propose a regularization term that makes our model more general and efficient for predicting unseen data. Also, it can provide learning with a small number of training data that is very useful for real applications. In the following, we first explain our regularization enhancement and then describe our cost-sensitive loss. Finally, we introduce how to use our cost-sensitive loss and regularization term with our proposed architecture.

#### Enhancing regularization in CapsNet

To train the capsule network, Sabor et al.^10^ proposed a new margin loss $L_k$ for *k* *th* class as Eq. (4):

4 $$\begin{aligned} L_k = T_k \,max(0, \,m^+ - \left\| \upsilon _k \right\| )^2 + \lambda (1-T_k) \,max(0, \,\left\| \upsilon _k \right\| - m^-)^2 \end{aligned}$$

where $T_k=1$ iff the input image belongs to the *k*th class, and $\left\| \upsilon _k \right\|$ indicates the activity of output vector $\upsilon _k$ for *k*th capsule in the final layer. We calculate the sum of the losses for all capsules in the final layer with the Eq. (5). Then the mean is taken over losses of all x in training batch X as follows:

5 $$\begin{aligned} L_{margin} = \frac{1}{N} \sum _{x=1}^{N} \sum _{k=1}^{K} L_{x, k} \end{aligned}$$

where *K* is the number of classes, and *N* is the number of training data or batch size in the current epoch. Sabour et al.^10^ and Hinton et al.^31^ added a reconstruction loss as a regularization term to the total loss for more generalization of the CapsNet and preventing overfitting. The reconstruction loss makes the network learn the object part and whole instantiation parameter so that the input image can be reconstructed well. In standard CapsNet, the image reconstruction is done using a decoder subnetwork consisting of some fully connected dense layers. To this end, the capsule values of the target class along with the masked capsules of the other classes are fed into the decoder to model the pixel intensities in which the mean of squared differences between the outputs and pixel intensities is minimized as Eq. (6):

6 $$\begin{aligned} L_{recons} = \frac{1}{N} \sum _{{\mathbf {x}} \in X}({\mathbf {x}}-\mathbf {d_{out}})^2 \end{aligned}$$

where $d_{out}$ is the output of the decoder. Finally, as Eq. (7), the total loss is the sum of the margin loss and the reconstruction loss where the reconstruction loss is scaled down by a factor of $\alpha$, which is near to zero hyper-parameter:

7 $$\begin{aligned} L_{total} = L_{margin} + \alpha \,L_{recons} \end{aligned}$$

hence, the Eq. (7) is used for backpropagation training of the standard capsule network. This new regularization makes the network model the main part of the input image that will reconstruct it. Consequently, it avoids paying attention to details for complex data that may lead to overfitting. With the best of our knowledge and experiments, the main disadvantage of regulation in standard CapsNet is when the input image size is large. In such a case, the dense reconstruction subnetwork requires many hidden neurons in each layer to obtain an appropriate image representation that is not applicable for today’s hardware. Ignoring the mentioned problem, training such a huge dense subnetwork requires lots of training data not available in many cases. The above limitations motivate us to propose a new regularization term for the capsule network. A weight tensor holds all the mapping matrices $W_{ij}$, which is used as the new regularization term in a capsule network. Therefore, the regularized loss is proposed as follows (8):

8 $$\begin{aligned} L_{total} = L_{margin} + \beta \left\| W \right\| _2 \end{aligned}$$

where $\beta$ is a hyper-parameter that controls the tensor norm, a positive close to zero real value, a detailed discussion about the effectiveness and implication of the regularized loss can be found in^30^.

#### Cost-sensitive loss

In the previous subsection, if the loss function is calculated by N for all samples or all batches, the tensor that contains all the losses is a member of $L\in {\mathbf {R}}^{N \times K}$, where K is the total number of classes. In the standard CapsNet, L is first added to the columns to obtain each sample’s loss to calculate the total loss function. The average of the resulting N vector is then calculated as the total loss in the backpropagation process. This computation is not always general; therefore, we propose a more efficient computation.

One of the main problems of averaging the loss function for all samples is to create an equal focus for the samples of all classes in the production process of the total loss function. In other words, in the face of class imbalance, given less minority class data available, network learning tends to minimize the loss of majority class data in the backpropagation process. To avoid the above problem, we propose to use of a cost-sensitive loss function. In this approach, instead of simply averaging *L* for all data, its weighted sum is used as follows:

9 $$\begin{aligned} L_{CS} = \frac{1}{N} \sum _{x_i \in X} w_i \sum _{k \in K} L_{ik} \end{aligned}$$

where N is equal to the total number of samples (i.e., number of batches) and $w_i$ is the weight of the *i*th sample. The weight of *i*th sample must be related to the inverse of the amount of data of its classmate. In other words, in the proposed method, the cost of incorrectly classifying data with a minority class is higher than the cost of classifying data with a majority class. As a heuristic, the following equation is used to calculate $w_i$:

10 $$\begin{aligned} w_i = \gamma - \frac{\left| Y=y_i\right| }{N_t} \end{aligned}$$

where $\left| Y=y_i\right|$ specifies the number of data labeled $y_i$ where $y_i$ is the label of data $x_i$ and $N_t$ is the total number of data in the training set. The variable $\gamma \ge 1$ is also a hyper-parameter for adjusting cost sensitivity. In other words, if the value of $\gamma>> 1$ is selected, the sensitivity of the loss function to the imbalance decreases. In this paper, the value of $\gamma = 1$ is used in implementations. In Jampour et al.^30^, a tensor norm that holds all mapping matrices is used as a regulator, and it has been shown that this method is effective in preventing the possibility of overfitting as well as the generalizability of the model. Therefore, we define the final loss function as:

11 $$\begin{aligned} L_{total} = L_{CS} + \beta \left\| W \right\| _2 \end{aligned}$$

where the hyper-parameter $\beta$ is equal to the regulatory coefficient and is a positive number close to zero.

## Results

### Datasets and protocols

This section provides various evaluations with different datasets and protocols to assess the proposed approach and show the method’s superiority. We employed two publicly available datasets HUST^32^ and COVID-CT-Dataset^33^ which are described briefly in the following.

#### HUST dataset

To prepare a standard and fair comparison, we followed the dataset and distribution of HUST, provided by Ning et al.^32^. This dataset contains 19,685 slice CT images in three categories of positive, negative, and non-informative for evaluation. The positive subset contains 4001 slice images, and the negative subset has 9979 images, while 5705 slice images are available in the non-informative class. For more details, please see the original paper^32^. In the following, we describe our employed protocols.

##### Protocol I

In the first protocol, we followed the data distribution of the original work, Ning et al.^32^, and used similarly 10-fold cross-validation, which means that we divided all classes into ten parts and used nine parts to train our model and one part as the test. We evaluate this procedure ten times where all data is considered the test sample and finally averaged all ten evaluations as the final result in this protocol.

##### Protocol II

Above, we followed the^32^ validation custom, but in many real-world problems, it is not realistic if we assume the positive and negative data are equal. Notably, in COVID-19, there are many negative data (CT images) even before the COVID-19 pandemic at the end of 2019. In contrast, there is a limited number of positive data, and therefore, imbalanced data is more realistic. Therefore, in the second protocol, we propose to show our approach superiority in imbalanced data. Thus, while the original data in protocol 1 contains 4001, 9979, and 5705 slice images for positive, negative, and non-informative classes, we first divide all data into two parts of training and test data with ratios 75% and 25%. Therefore, we have 3000, 7484, and 4278 positive, negative, and non-informative images for the training part and 1001, 2495, and 1427 positive, negative, and non-informative images for the test part. We then define new imbalance protocols on training data, including 1500, 750, 500, 300, 250, 187, and 150 positive CT slice images with the same negative and non-informative images. With this amount of positive data our imbalance ratio (IR) defines $IR \approx 5$ ($\frac{7484}{1500}$), 10, 15, 25, 30, 40, and 50 ($\frac{7484}{150}$) respectively. Of course, it is crucial if an approach achieves reasonable accuracy in the presence of a low number of positive data. Our motivation is to show our approach feasibility in only 150 positive CT slice images in the last imbalanced evaluation. We also note that we selected the first N positive CT slice images from the whole 3000 training images (e.g., the first 150 positive images for the last evaluation). Obviously, the bigger IR is more difficult for the model to learn for finding COVID-19 pathologies in the lung.

#### COVID-CT dataset

To show the generalization ability of our approach, we provided more experiments on another popular dataset, namely, COVID-CT Dataset^33^. The COVID-CT Dataset is a small dataset with two categories of COVID and non-COVID images, which were recently used in similar techniques. Note that in this dataset, non-COVID images contain other diseases such as lung cancer, etc. We used this dataset to provide another comparison with the related works. This dataset has 349 positive COVID images from 216 patients. Inspired from related works^17,24,26,34^ we followed the k-fold validation with $k=4$, $k=5$, and $k=10$ to provide fair comparisons. The results and more details on this dataset are provided in “Evaluation on COVID-CT”.

### Pre-processing

As the dataset images are simple CT output images, they need pre-processing to extract the lung area better. However, the pre-processing influence is negligible. Instead, it helps extract the region of interest (ROI) and provides a well-formed and user-friendly deep architecture. For this purpose, we first converted the images to grayscale format. After using simple thresholding, we applied the well-known morphological operations, including filling, closing, and opening, to all of them. The obtained areas were analyzed after morphological operations, and small areas or areas which were not elliptic in shape were removed. Finally, the lung’s detected area is reshaped into a square image and then resized to the standard size $224 \times 224$ and feed into our proposed network as input data.

### Evaluation on HUST—Protocol I

According to Table 1, we developed three methods to solve the early COVID-19 detection using CT images. The first uses basic DenseNet, which is an efficient deep approach for feature (pathology) description. The second developed method is our hybrid version of DenseNet with CapsNet that benefits both networks’ advantages. We also enhanced this hybrid architecture in terms of regularization, and therefore, as can be seen, its results are better than the basic DenseNet. Finally, the third method, a very efficient and successful deep approach for COVID-19 early detection, is our enhanced method with a novel Cost-sensitive loss function that outperformed all the previous techniques even in imbalanced conditions. We compared our developed methods with the same dataset and protocol as proposed in the original paper^32^. Figure 3 in the main paper shows that the accuracy of each class of NiCT, pCT, and nCT is 91.97%, 92.38%, and 99.42%, while the number of samples in these classes are 5705, 4001, and 9979, respectively. Therefore, it is possible to compute that the original paper’s overall accuracy is 95.83%. We compute and report our methods’ results in terms of Sensitivity, Specificity, and Accuracy in Table 1 with the same protocol to perform a fair comparison. As can be seen, all our methods’ accuracy is significantly better than the original work in all classes. Also, our regularized CapsNet conjugated with DenseNet (RegCapsDenseNet) expectedly outperforms the standard DenseNet, and the Ning et al.^32^. Our proposed regularized Cost-sensitive CapsNet conjugated with DenseNet (RegCS.CapsDenseNet) gains higher sensitivity and accuracy than previous methods.

**Table 1:** Performance evaluation (%) of our developed deep networks on the HUST dataset compared to the original paper.

Dataset	Type	Accuracy	Sensitivity	Specificity
Original paper^32^	NiCT	91.97	97	90.68
	pCT	92.38	85.47	99.12
	nCT	99.42	98.40	99.64
Standard DenseNet	NiCT	98.80	99.90	98.54
	pCT	98.80	97.65	99.96
	nCT	100	100	100
RegCapsDenseNet	NiCT	99.78	99.14	99.94
	pCT	99.78	99.91	99.65
	nCT	100	100	100
RegCS.CapsDenseNet	NiCT	**99.89**	99.66	99.94
	pCT	**99.89**	99.91	99.87
	nCT	**100**	100	100

The highest accuracy is shown in bold.

### Evaluation on HUST—Protocol II

As explained before, there is a limited number of positive COVID-19 slice CT images compared to negative in the real-world condition. Therefore, we proposed a Cost-sensitive loss function for our deep hybrid network. To show the impact of our main idea for imbalanced data, we used different numbers of positive images with constant negative CT images. To this end, we define the term of Imbalance Ratio (IR) as IR = $\frac{N_s}{P_s}$ where $N_s$ and $P_s$ show the number of negative and positive samples, respectively. It is clear that more IR value means a much more challenging condition for deep learning approaches due to the low number of positive data compared to the negative samples or imbalanced situations. Therefore, we expect lower accuracy for bigger IR. Nevertheless, as shown in Table 2, our proposed regularized Cost-sensitive (RegCS.CapsDenseNet) approach has better accuracy than our regularized (RegCapsDenseNet) method and also the standard DenseNet in all imbalanced situations. It is expectable that the final accuracies decreased with increasing the IR; nevertheless, our RegCS.CapsDenseNet is more stable in the presence of imbalanced data that is more visible in bigger IR evaluations.

**Table 2:** Performance evaluation (%) of our developed deep networks on the HUST dataset in various imbalanced conditions

Approach	Measure	IR = 5	IR = 10	IR = 15	IR = 25	IR = 30	IR = 40	IR = 50
Standard DenseNet	Precision	99.90	99.80	99.79	100	99.78	100	100
	Recall	99.70	99.20	95.60	92.00	91.20	89.80	83.20
	Accuracy	99.92	99.80	99.07	98.37	98.17	97.92	96.59
RegCapsDenseNet	Precision	100	100	100	100	100	100	99.35
	Recall	99.60	99.30	97.40	96.70	94.00	93.30	92.40
	Accuracy	99.92	99.86	99.47	99.33	98.78	98.64	98.33
RegCS.CapsDenseNet	Precision	100	100	100	99.9	100	100	99.36
	Recall	99.70	99.40	98.10	97.40	94.80	94.70	93.00
	Accuracy	**99.94**	**99.88**	**99.61**	**99.45**	**98.94**	**98.92**	**98.46**

The highest accuracy is shown in bold.

**Figure 2:**
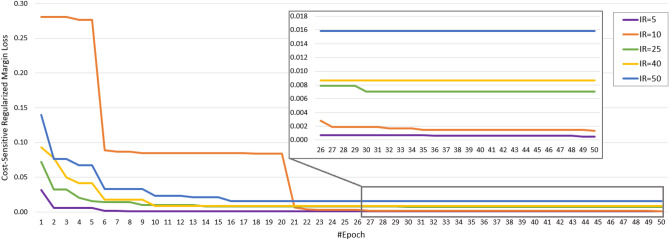
The Cost-sensitive regularized margin loss (error) of our RegCS.CapsDenseNet method with different imbalance ratios (IR) during the learning process on the HUST dataset.

**Figure 3:**
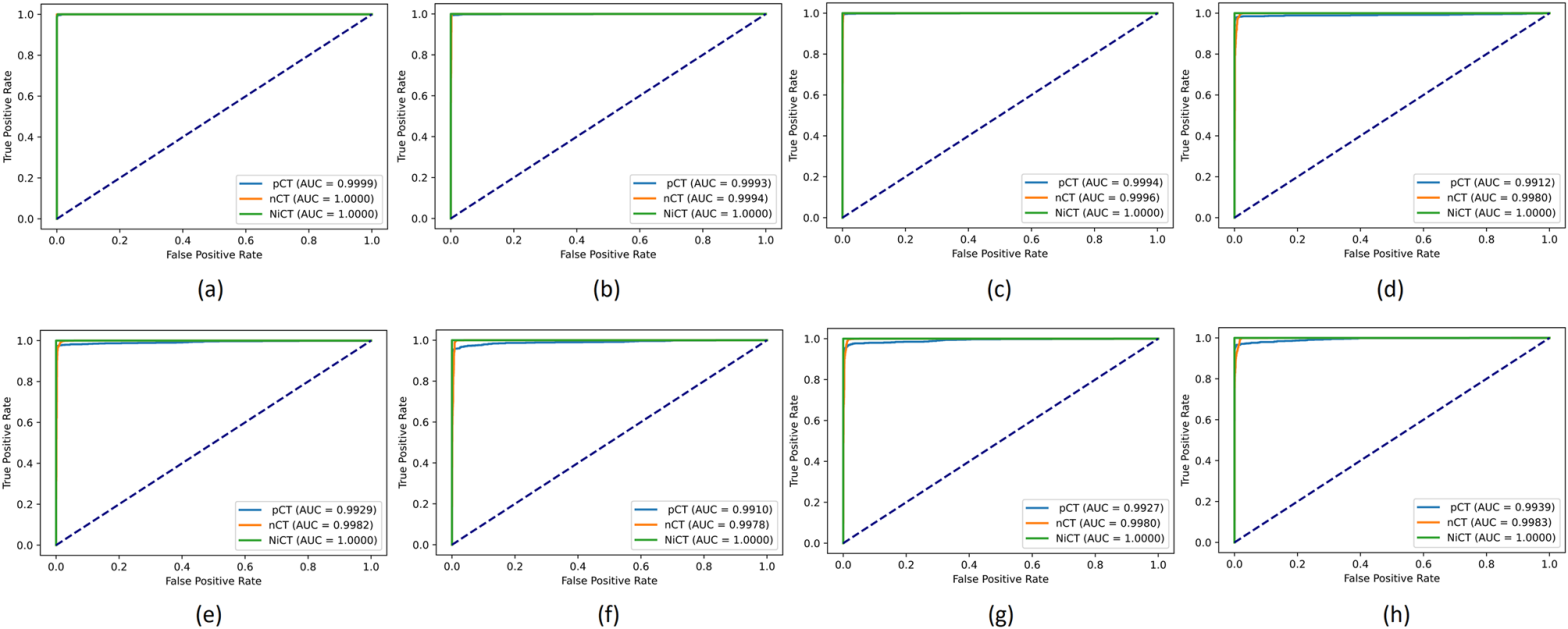
ROC curves generated by our technique on dataset HUST. (**a**) Protocol I, (**b**–**h**) Protocol II with different imbalance ratios (IR) = 5, 10, 15, 25, 30, 40, and 50, respectively.

**Figure 4:**
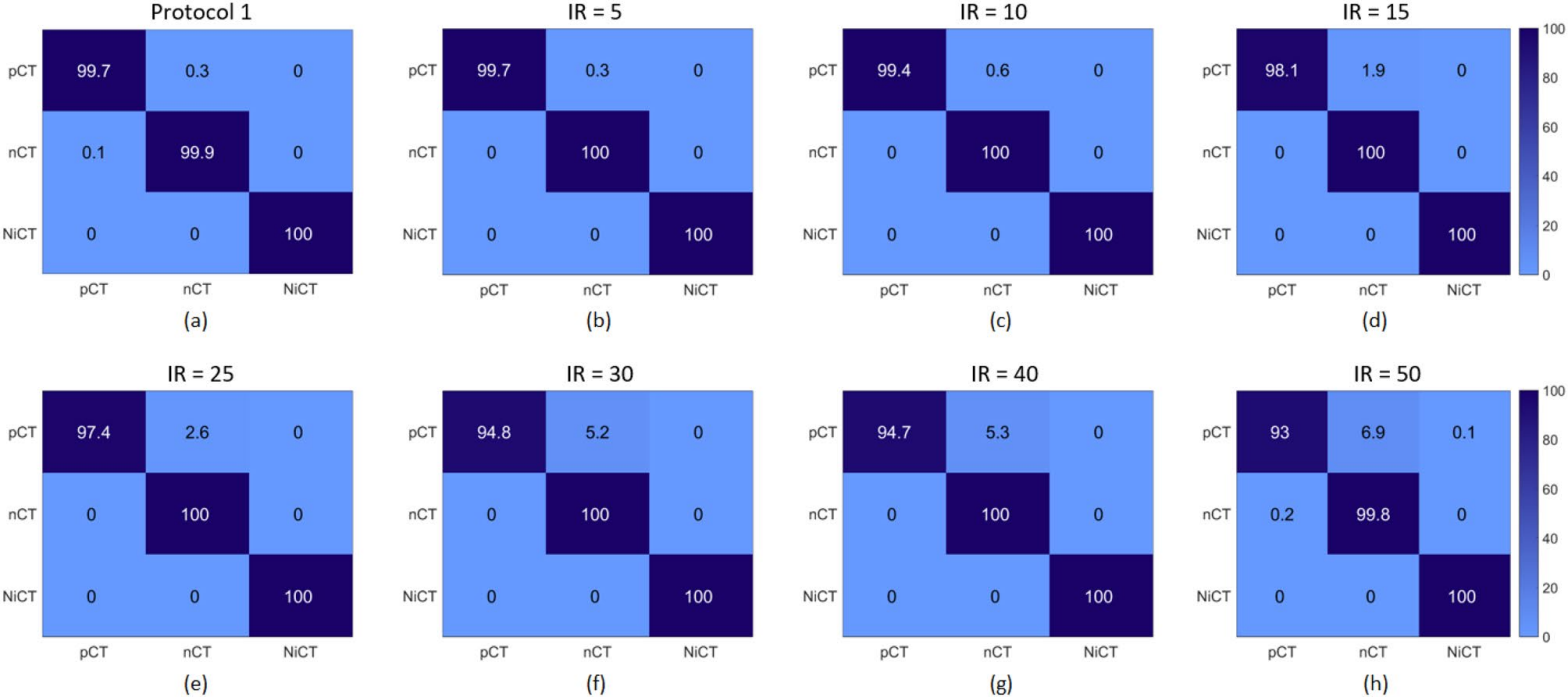
The confusion matrices of our RegCS.CapsDenseNet method. (**a**) with the first protocol. (**b**–**h**) the second protocol with different imbalance ratios (IR) = 5, 10, 15, 25, 30, 40, and 50, respectively.

Besides, to provide a much more reasonable evaluation, we used an F1-score measure that considers precision and recall. The highest value indicates perfect precision and recall, which is desirable in medical image analysis. F1-score computes as $F_1 = \frac{2 \times precision \times recall}{precision + recall}$. Table 3 shows the F1-score evaluation of our methods and, as can be seen, our enhanced RegCS.CapsDenseNet has a better score in all IR situations in all classes. Moreover, there is a considerable difference in F1-Score between IR = 5 and IR = 50 of standard DenseNet, whereas the difference is subtle for the proposed RegCS.CapsDenseNet. Furthermore, to show the impact of imbalanced data, Fig. 2 shows the Cost-sensitive regularized margined loss for our RegCS.CapsDenseNet method in the presence of imbalanced data. However, we expect a bigger loss for a bigger IR. Still, our approach is almost stable with enough learning processes. The training error is significantly reduced at the beginning of epochs and continues with a smoother slope in the following training epochs. We also generated the Receiver Operating Characteristic (ROC) curves obtained by our RegCS.CapsDenseNet method in Fig. 3 where the ROC curve of HUST dataset with its Area Under the Curves (AUC) rates is shown in subfigure (a), and the curves in (b) to (h) are regarding the protocol II with different IRs. In the ROC curve, the point of (0, 1) based on the Cartesian plane indicates the highest sensitivity and specificity; therefore, as can be seen, our method obtained a reasonable classification rate. Finally, to show the correlation between classes, we provided our evaluation confusion matrices in Fig. 4. In this figure, the confusion matrix (a) is related to the first protocol, and comparable with the original work^32^. Other remained seven confusion matrices (b) to (h) are related to the seven different IRs in the second protocol where (b) is for IR = 5, and (h) shows the confusion matrix for IR = 50. We can find that the most confusion happens between positive and negative classes from all the confusion matrices.

**Table 3:** F1-Score evaluation (%) of our developed deep networks on the HUST dataset in various imbalanced conditions.

Approach	Type	IR = 5	IR = 10	IR = 15	IR = 25	IR = 30	IR = 40	IR = 50
Standard DenseNet	pCT	99.80	99.50	97.65	95.83	95.30	94.63	90.83
	nCT	99.92	99.80	99.09	98.42	98.23	98.00	96.80
	NiCT	100	100	100	100	100	100	99.89
RegCapsDenseNet	pCT	99.80	99.65	98.68	98.32	96.91	96.53	95.75
	nCT	99.92	99.86	99.52	99.34	98.81	98.71	98.40
	NiCT	100	100	99.93	100	100	99.93	99.96
RegCS.CapsDenseNet	pCT	99.85	99.70	99.04	98.63	97.33	97.28	96.07
	nCT	99.94	99.88	99.62	99.46	98.95	98.95	98.52
	NiCT	100	100	100	100	100	100	99.96

### Evaluation on COVID-CT

**Figure 5:**
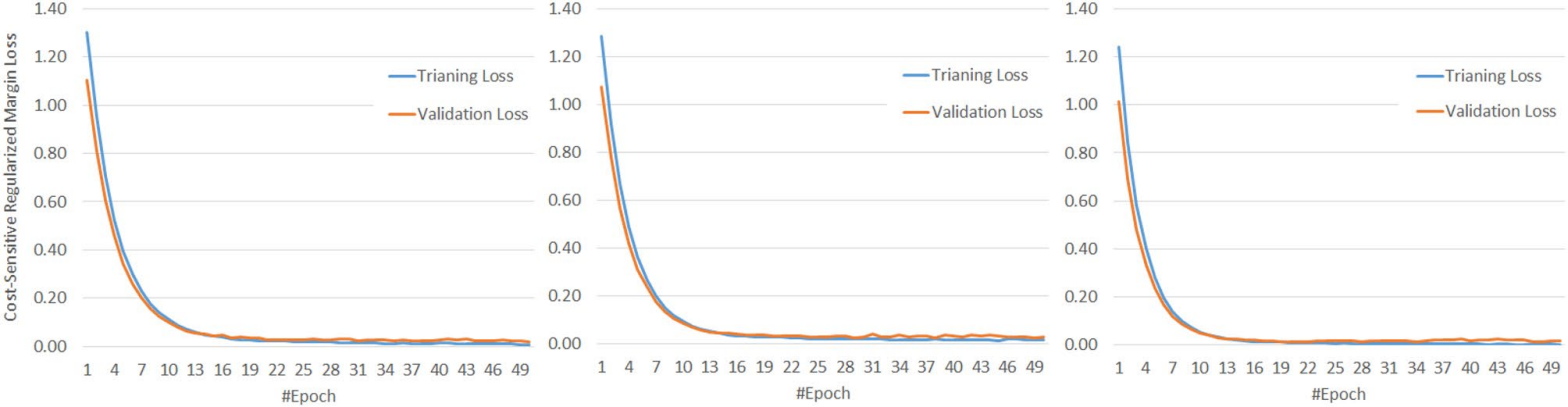
The cost-sensitive regularized margin loss (error) of our RegCS.CapsDenseNet method with three k-fold validations. (left) k = 4, (center) k = 5, and (right) k = 10 on the COVID-CT Dataset.

This section emphasizes comparison with the related works using standard metrics such as Accuracy, Precision, Recall/Sensitivity, Specificity, and F1-score. To this end, we employed the COVID-CT dataset as described in “COVID-CT dataset”, which is a popular dataset in related works. However, there is no unique setup for this dataset due to the lack of a standard protocol. Therefore, we provided three different k-fold validations with $k=4$, $k=5$, and $k=10$ in Table 4, the average and standard deviation of each metric are reported for better comparison. As can be seen, the results of our approach in terms of Precision, Recall, Specificity, and Accuracy outperformed significantly the state-of-the-art^17,21,34^ in both 4-fold and 10-fold. In 5-fold, we achieved reasonable results; however, our average accuracy is less than the results of Pham et al.^26^. Nevertheless, the standard deviation of our results compared to their approach shows that our proposed approach is much more stable than their technique regarding input data distribution. In addition, as shown in Fig. 5, the training loss significantly decreases during epochs as well as the loss curve for the validation data. Therefore, the learning occurred well during epochs, and the network converges aimed fast, especially in the earlier epochs. Finally, we note that we did not use augmentation in all experiments to show our approach capability of recognizing COVID-19 pneumonia with a low number of data. This capability of working with a low number of data is a desirable goal for deep approaches that need lots of data.

**Table 4:** Comparison of our proposed RegCS.CapsDenseNet model with related works on COVID-19 dataset with different validations.

Method	Val.	Accuracy (%)	Precision (%)	Recall/Sens (%)	Spec (%)	F1-score (%)
Single model^34^	4-fold	$77.07 \pm 1.92$	$79.48 \pm 0.96$	$74.69 \pm 3.91$	NA	$77.04 \pm 2.17$
MS-Net^34^	4-fold	$76.23 \pm 1.81$	$79.29 \pm 1.48$	$74.07 \pm 1.29$	NA	$76.54 \pm 1.73$
SepNorm + Contrastive^34^	4-fold	$78.69 \pm 1.54$	$78.02 \pm 1.34$	$79.71 \pm 1.42$	NA	$78.83 \pm 1.43$
**Ours (RegCS.CapsDenseNet)**	4-fold	**89.40 ± 2.10**	**89.04 ± 4.36**	**88.22 ± 2.71**	**90.48 ± 3.83**	**88.57 ± 2.47**
EfficientNet-B3 – Arc1^24^	5-fold	83.74	NA	83.67	NA	83.25
EfficientNet-B3 – Arc2^24^	5-fold	87.68	NA	79.59	NA	86.19
AlexNet^26^	5-fold	$86.85 \pm 13.7$	NA	$80.25 \pm 22.49$	**94.29 ± 4.84**	$85 \pm 16$
SqueezeNet^26^	5-fold	$87.52 \pm 6.45$	NA	$86.84 \pm 10.11$	$88.29 \pm 12.01$	$88 \pm 6$
NasNet-Large^26^	5-fold	$88.59 \pm 7.59$	NA	$90.51 \pm 0.90$	$86.43 \pm 17.17$	$90 \pm 6$
Inception-ResNet-v2^26^	5-fold	$88.59 \pm 7.59$	NA	$89.24 \pm 2.69$	$87.86 \pm 13.13$	$96 \pm 5$
NasNet-Mobile^26^	5-fold	$89.26 \pm 8.14$	NA	$91.56 \pm 5.12$	$86.67 \pm 13.27$	$95 \pm 6$
**Ours (RegCS.CapsDenseNet)**	5-fold	$89.81 \pm 1.76$	$88.97 \pm 2.11$	$89.11 \pm 3.09$	$90.39 \pm 0.99$	$89.02 \pm 2.17$
Xception^26^	5-fold	$91.11 \pm 10.1$	NA	$89.56 \pm 12.55$	$92.86 \pm 7.80$	$91 \pm 10$
ResNet-18^26^	5-fold	**95.44 ± 8.02**	NA	**98.99 ± 1.65**	$91.43 \pm 15.25$	**96 ± 7**
Custom CNN^17^	10-fold	84.56	80.24	88.23	81.44	83.98
xDNN^21^	10-fold	88.60	89.70	88.60	NA	89.20
**Ours (RegCS.CapsDenseNet)**	10-fold	**92.49 ± 2.87**	**91.67 ± 4.06**	**92.30 ± 5.80**	**92.66 ± 3.63**	**91.86 ± 3.47**

The highest values in each protocol are shown in bold.

## Discussion and future works

Over the past year, all countries have been infected with pneumonia COVID-19, and the lives of millions of people have been endangered by the coronavirus. Diagnosis of the disease in the early stages to reduce cross-infection and stop the disease’s progression is very important. Besides, the automated analysis of CT images of the patient is critical in the early stages of the outbreak to prevent the further spread of the infection. It can also help physicians decide on the type and duration of treatment or isolation and other necessary status checks. Using the CT images is one of the most common modalities available for screening and clinical diagnosis of COVID-19 pneumonia. Moreover, in recent days, the workload of radiologists is massive daily, so a patient must be awaited several hours for a CT scan report. Also, expert radiologists in thoracic imaging may not be available at every institution. All of these factors increase the risk of cross-infection. Recently, deep learning has been introduced as a powerful tool for many image-related problems, including COVID-19 pneumonia detection. This paper proposed a deep learning-based framework that can extract valuable features from the CT image. Our method achieves an accuracy of 99.95% for COVID-19 early detection compared to 95.83% of Ning et al.^32^. We achieved 99.89%, 99.89%, and 100% accuracy for NiCT, pCT, and nCT classes while the original paper by Ning et al.^32^ reported 91.97%, 92.38%, and 99.42% for the same classes with the same protocol. Moreover, to address the capability of our approach even in the presence of a low number of training data, we provided more experiments with the COVID-CT dataset with three different k-fold validations. The proposed method gains higher Accuracy, Precision, and Recall compared to state-of-the-art methods^17,21,24,34^ except results reported by^26^. However, the dispersion of our results compared to their approach shows that our approach is much more stable than their technique regarding input data distribution. This result shows our method superiority for involved pathologies exploration and our method formulation effectiveness.

Although a recently automatic diagnosis of COVID-19 pneumonia has been introduced with the deep learning methods, there are still many unsolved challenges in the automatic and analysis of CT images related to this epidemic. The lack of public and large datasets is an important challenge for this application so that a very recent approach used data augmentation^35^ or applied GAN^12^, or its extension, Cycle-GAN (CGAN)^13^ to reduce the gap of needs to the lots of data. Also, the main problem of existing public datasets is that all taken images are labeled based on the patient. However, when imaging the patient’s lungs, several cuts are taken (i.e., between 50 and 150) where all of them must not be included with the pathology. Therefore, each image must be separately labeled by the physician. When training the network, images with the appropriate label are available, and the network is appropriately trained to perform slice-level classification. There is also a lack of access to datasets that contain clinical features in addition to the CT image that can be used to predict mortality, disease side effects, disease severity evaluation, and other clinical usages. Learning both image information and clinical information can lead to better network performance. For instance, very recently, Lassau et al.^36^ integrated clinical and radiological data to predict the severity of COVID-19. Another problem with datasets is the imbalance of images. As COVID-19 is a new epidemic that encountered humans for about a year, other lung-related epidemics, including cancers and other viral pneumonia such as influenza, have been around for a long time. Therefore, there is a rich collection of images related to these diseases. However, deep learning methods have good potential for diagnosing COVID-19^3,32^, the condition with a limited number of positive data is not considered. In this study, we focused on the imbalanced data challenge and proposed a novel cost-sensitive loss function to overcome it.

### Limitations and future works

While our framework provides a very efficient outcome regarding the COVID-19 pneumonia recognition and can also process large images even with a small number of training data, it still has limitations. For instance, as input CT images are of various sizes and qualities, we need to consider a pre-processing stage before analyzing them. Of course, it is desirable to provide our system to input CT images of any size or any resolutions. Also, as the CT images have 3-dimensions (slices) intrinsically, it is desirable to propose an end-to-end Capsule network model capable of analyzing them. Moreover, the noticeable point is that many pieces of research introduced for COVID-19 diagnosis, including our study, whereas just provided with the image and the associated label, their output does not contain any explicit segmentation of diagnostically helpful components. Proposing methods that are more sensitive to the classification and segmentation of images, especially with minor lesions, are considered very important for the early COVID-19 diagnosis. In the early stage of the disease, pathologies are usually subtle and difficult to detect. It is also exciting to segment the pneumonia regions to approximate a measure of Coronavirus involvement or progress. To this end, quite recently, works such as^37^ try to obtain segmentation maps for these lesions. Lesions segmentation and measuring to evaluate the progress could be a direction for future studies.

## Conclusion

Recently, deep learning-based approaches have shown very successful performance to help physicians or reduce doctors’ workload for COVID-19 detection from CT images. Most of these approaches are based on deep neural networks (DNNs) techniques. Nevertheless, a significant drawback of the deep methods is their need for many training samples, which are not always accessible. Besides, in the case of the availability of COVID-19 CT images, there are typically imbalanced data due to the limited number of positive samples. In this work, we proposed an efficient hybrid deep architecture, including DenseNet and CapsNet, to which we benefit both networks’ advantages. Also, we proposed a novel Cost-sensitive loss function to overcome imbalanced data. Our results show that the proposed idea leads to a very robust design in the presence of imbalanced data even if the positive samples are 50 times less than the negative data. Moreover, we enhanced our model with a regularization term that makes our model more general and efficient in reducing the learning parameters and memory usage. We evaluated our approach on two publicly available datasets HUST and COVID-CT, with various protocols. In the first protocol of HUST, we followed the original paper setup that contains 4001, 9979, and 5705 images for positive, negative, and non-informative classes, respectively. Our proposed approach outperformed it by achieving 99.95% accuracy. The second protocol that we used is based on the imbalanced data condition (N-first positive images, where N is 150, 187, 250, 300, 500, 750, and 1500) to show our approach superiority in the presence of a limited number of positive data. In imbalanced data, we achieved 98.46%, 98.92%, 98.82%, 99.45%, 99.61%, 99.88%, and 99.94% accuracy, respectively that show our approach robustness when the data is imbalanced. Moreover, we evaluated our approach with three different k-fold validations on the COVID-CT dataset where $k=4$, $k=5$, and $k=10$ to compare the related works. Our proposed method achieved $89.40 \pm 2.10$, $89.81 \pm 1.76$, and $92.49 \pm 2.87$ accuracies, which is better than the state-of-the-art in 4-, and 10-fold.

## Data Availability

No datasets are generated during the current study and the used datasets are publicly available.

## Code availability

Our source codes are publicly available for research purposes in the GitHub repository: https://github.com/Javidi31/COVID19_Reg_Caps_Dense/tree/master.
